# *Vibrio* pore-forming leukocidin activates pyroptotic cell death via the NLRP3 inflammasome

**DOI:** 10.1080/22221751.2020.1720526

**Published:** 2020-02-04

**Authors:** Hadar Cohen, Noam Baram, Liat Edry-Botzer, Ariel Munitz, Dor Salomon, Motti Gerlic

**Affiliations:** Department of Clinical Microbiology and Immunology, Sackler Faculty of Medicine, Tel Aviv University, Tel Aviv, Israel

**Keywords:** Cell death, inflammasome, pyroptosis, IL-1β, *Vibrio*, leukocidin, hemolysin, pore-forming

## Abstract

Cell death mechanisms are central to combat infections and to drive inflammation. The inflammasome controls infection through activation of caspase-1 leading to either IL-1β dependent inflammation, or pyroptotic cell death in infected cells. Hemolysins, which are pore-forming toxins (PFTs), alter the permeability of the host target membrane, often leading to cell death. We previously discovered a leukocidin domain-containing PFT produced by the Gram-negative bacterium *Vibrio proteolyticus*, named VPRH. VPRH constitutes a distinct, understudied class within the leukocidin superfamily, which is distributed among several photogenic *Vibrios*. Since PFTs of other pathogens were shown to activate the inflammasome pathway, we hypothesized that VPRH-induced cell death is mediated by direct activation of the inflammasome in mammalian immune host cells. Indeed, we found that VPRH induced a two-step cell death in macrophages. The first, a rapid step, was mediated by activating the NLRP3 inflammasome, leading to caspase-1 activation that resulted in IL-1β secretion and pyroptosis. The second step was independent of the inflammasome; however, its mechanism remains unknown. This study sets the foundation for better understanding the immunological consequences of inflammasome activation by a new leukocidin class of toxins, which may be shared between marine bacteria and give rise to new pathogenic isolates.

## Introduction

Pathogenic bacteria often produce and secrete toxins to manipulate their hosts or to defy predators. A widespread and abundant class of virulence factors are pore-forming toxins (PFTs) [1]. Pore-forming hemolysins oligomerize in the plasma membrane of the target cell and produce pores with different diameters; these pores alter the permeability of the target plasma membrane to small molecules and even proteins, often leading to cell death.

Innate immune responses combat infectious microbes, but may also drive pathological inflammation. Cell death mechanisms are central for these processes, since they lead to the death of an infected cell, as well as to the release of danger-associated molecular patterns (DAMPs) that drive the inflammatory process in either a beneficial or pathological manner [2]. A cell death mechanism, known as pyroptosis, was shown to be important for controlling several infections by activating the inflammasome complexes and the subsequent secretion of the mature pro-inflammatory cytokines, interleukin 1β (IL-1β) and IL-18 [3,4].

Inflammasomes are multimeric protein complexes that assemble in the cytosol after sensing pathogen-associated molecular patterns (PAMPs) or DAMPs. Their activation is mediated by evolutionarily conserved innate immune pattern recognition receptors (PRRs) [5]. Inflammasomes can be divided into 2 categories: canonical, in which procaspase-1 is converted into a catalytically active enzyme [6], and noncanonical, which are initiated by the activation of caspase-11 [7]. The canonical inflammasome contains a nucleotide-binding oligomerization domain (NOD), leucine-rich repeat (LRR)-containing protein (NLR) family member (e.g. NLRP1, NLRP3, or NLRC4), or the DNA sensor, Absent in Melanoma 2 (AIM2). NLRs and AIM2 contain a pyrin domain (PYD) or a caspase recruitment domain (CARD) [4] that interacts with the apoptosis-associated speck-like protein containing a CARD (ASC) adaptor or with procaspase-1, directly. This interaction leads to dimerization, autocleavage, and activation of caspase-1 [8], which further cleaves the inactive precursors of IL-1β and IL-18 into their active, pro-inflammatory forms, and directs their secretion; it may also lead to pyroptotic cell death [9,10].

We previously discovered a functional and potent pore-forming hemolysin produced by the Gram-negative marine bacterium *Vibrio proteolyticus* (*V. proteolyticus*), termed VPRH (*Vibrio proteolyticus* hemolysin) [11]. *V. proteolyticus*, which was first isolated from the gut of the wood borer *Limnoria tripunctata* [12], is pathogenic to marine animals; it was isolated as part of a *Vibrio* consortium from yellow band diseased corals [13], and was shown to cause mortality in fish [14] and in the crustacean model organism, *Artemia* [15]. The hemolysin, VPRH, is a 305-residue protein containing a secretion signal peptide followed by a leukocidin domain. Although the VPRH leukocidin domain is homologous to those found in other known PFTs, such as α-hemolysin, HlyA, and LukED, VPRH defines a distinct and understudied class within the leukocidin superfamily [11]. Members of the VPRH leukocidin class are confined to marine bacteria, including emerging pathogens of humans and marine animals. Recently, we showed that when VPRH was introduced to cultures of human epithelial HeLa cells, it caused changes in the actin cytoskeleton, resulting in cell lysis. Similar VPRH-dependent lytic activity was also found when *V. proteolyticus* bacteria were added to murine RAW 264.7 macrophage cell cultures [11].

A common result of PFT insertion into the plasma membrane is a drop in cellular potassium concentration, which leads to activation of signaling cascades such as the inflammasome and mitogen-activated protein kinase pathways [16]. Several pore-forming leukocidins, such as *Staphylococcus aureus* α-hemolysin and Panton-Valentine leukocidin [17], were found to affect inflammasome activation in mammalian immune cells. Since VPRH was only tested against cells that do not possess a functional inflammasome (HeLa and RAW 264.7), it is not known whether members of the VPRH class of leukocidins affect immune cells similarly.

In this work, we sought to determine whether VPRH affects the inflammasome, and if so, to decipher the underlying mechanism. Importantly, we found that VPRH induced a rapid cell death in bone marrow-derived macrophages (BMDMs), in comparison with the slower cell death induced in HeLa and RAW 264.7 cells that do not contain a functional inflammasome [8]. Using chemical inhibitors, we determined that the cell death in BMDMs comprised two distinct steps: the first, a rapid step, was pyroptosis; while the mechanism underlying the second, a slower step, remains unexplored. Furthermore, we demonstrated that VPRH-induced pyroptosis was dependent on the NLRP3 inflammasome, since NLRP3-deficient BMDMs were protected from the initial, rapid cell death. In agreement with these findings, VPRH led to the specific secretion of the pro-inflammatory cytokine IL-1β in a NLRP3-dependent manner in BMDMs and human peripheral blood mononuclear cells (PBMCs). Therefore, we concluded that VPRH induces cell death in mammalian cells; this cell death is accelerated in primary macrophages by rapid activation of the NLRP3 inflammasome and pyroptosis.

## Materials and methods

### Reagents

Unless stated otherwise, all cell culture reagents were purchased from Biological Industries, Beit-Haemek, Israel. Lipopolysaccharides (LPS) of *Escherichia coli* O111:B4 were purchased from Sigma-Aldrich (#L3024). Propidium Iodide (PI) was purchased from Sigma-Aldrich (#P4170). Necrosulfinamide (NSA) was purchased from Tocris Bioscience; Vx765 and MCC950 were purchased from Invitrogen. HRP-conjugated secondary antibodies were purchased from Jackson ImmunoResearch Labs (West Grove, PA, USA). ELISA kits were purchased from eBioscience or R&D.

### Mice

C57/BL6/J (wild-type [B6J]), Nlrp3A350VneoR/+, which are NLRP3 KO [18], NLRP1 KO [10], and MLKL KO [19] mice, were bred under specific pathogen-free conditions in the animal facility at Tel Aviv University. Experiments were performed according to the guidelines of the Institutes’ Animal Ethics Committee.

### Cell culture

PBMCs and HeLa, RAW 264.7, and BMDM cells were grown in DMEM culture medium containing 10% FBS, 1% penicillin–streptomycin, and 1% HEPES, at 37°C, in a 5% CO_2_ incubator.

### Bone marrow-derived macrophages

Bone marrow (BM) cells from mice were isolated by flushing femurs and tibias with 5 ml PBS, supplemented with 2% heat-inactivated fetal bovine serum (FBS) Gibco (Thermo Fisher Scientific, Waltham, MA, USA). The BM cells were centrifuged once and then re-suspended in tris-ammonium chloride at 37°C for 30 s to lyse red blood cells. The cells were centrifuged again and then strained through a 70 μm filter before being re-suspended in DMEM supplemented with 10% FBS. BMDMs were obtained by 7 days differentiation with L-con media as previously described [20].

### Peripheral blood mononuclear cells (PBMCs)

PBMCs used in infection studies were obtained from healthy donors and isolated by density-gradient centrifugation using Histopaque-1077 (Sigma-Aldrich, 10771), as previously reported [21]. Briefly, 10 ml peripheral blood were collected from three individual healthy donors. Blood was diluted 1:2 in PBS, loaded on Histopaque-1077, and centrifuge for 30 min at 400 g at 24°C. Cells from the interphase were collected and washed with PBS. Sample of cells were then stained for flow cytometry to determine the percentage and concentration of monocytes, before they were seeded in a 96-well plate at a final concentration of 1.75*10^5^/ml in triplicate in 1% FBS and penicillin–streptomycin-free DMEM. After 18 h, the wells were washed once to remove non-adherent cells, and adherent cells were used for subsequent infection experiments. Experiments were performed according to the guidelines of the Institute's Helsinki Ethics Committee.

### Bacterial strains and media

*Vibrio proteolyticus* strain ATCC 15338 (also termed NBRC 13287) and its derivatives were routinely grown in Marine Lysogeny Broth (MLB; Lysogeny broth supplemented with NaCl to a final concentration of 3% w/v) at 30°C. To induce the expression of genes from a plasmid, 0.1% (w/v) L-arabinose was included in the media. When necessary to maintain plasmids, the media were supplemented with 250 µg/ml kanamycin. Construction of the *vprh* (locus tag VPR01S_RS09275; encoding WP_021705060.1) deletion strain (Δ*vprh*) and of the VPRH arabinose-inducible expression plasmid (pVPRH) were reported previously [11].

### Bacterial growth assay

Overnight-grown cultures of *V. proteolyticus* were normalized to an OD_600_ = 0.01 in MLB media and transferred to 96-well plates (200 µl per well). For each experiment, *n* = 3. Cultures were grown at 30°C in a BioTek EPOCH2 microplate reader with continuous shaking at 205 cpm. OD_600_ readings were acquired every 10 min. Experiments were performed at least three times with similar results.

### Bacterial swimming assay

Swimming media plates were prepared with Lysogeny broth containing 20 g/l NaCl and 3 g/l Agar. *V. proteolyticus* strains that were grown overnight on a MLB plate were picked and then stabbed into the swimming plates using a toothpick (*n* = 3). Plates were incubated at 30°C for 8–16 h. Swimming was assessed by measuring the diameter of the spreading bacteria. Experiments were performed three times with similar results.

### Infection of cell cultures

Unless otherwise stated, PBMCs, and HeLa, RAW 264.7 and BMDM cells were washed 3 times using PBS, and then seeded at a final concentration of 1.75*10^5^/ml in triplicate in 1% FBS and penicillin–streptomycin-free DMEM. Cells were infected with *V. proteolyticus* mutants at MOI 20 or 50, as indicated. Where indicated, BMDMs were pre-incubated with LPS (100 ng/ml, 3 h). When used, inflammasome inhibitors Vx765 (25 μM), MCC950 (20 μM), and NSA (20 μM) were added 30 min prior to infection. More specifically, overnight cultures of *V. proteolyticus* mutants were diluted and prepared in DMEM without antibiotics. Bacteria were added to the wells, and plates were centrifuged for 5 min at 400 g. Plates were then inserted into IncucyteZOOM for incubation and for monitoring cell death.

### Live cell imaging

Plates with infected cells were placed in IncucyteZOOM (Essen BioScience) and images were recorded every 10–30 min. Data were analyzed using IncucyteZoom2016B analysis software and exported to GraphPad Prism software. Normalization was then performed according to the maximal PI-positive object count to calculate the percentage of dead cells.

### Immunoblot analyses of proteins

Cells were collected and centrifuged for five minutes at 400xg (4°C) in order to separate them from the supernatant. Next, the cells were lysed either by using RIPA buffer in the presence of protease inhibitors at 4°C for 15 min, or directly by applying denaturing western blot sample buffer (SDS*1) to cells. Lysed cells were loaded onto any kD gradient Criterion TGX-Free precast gels (Bio-Rad). Proteins were transferred onto a nitrocellulose membrane (Bio-Rad), and Ponceau S staining was performed routinely to evaluate the loading accuracy. Membranes were blocked with 5% (w/v) skim milk in TBS for 1–2 h, and then probed overnight with primary antibodies (all diluted 1:1000, unless noted otherwise): mouse-NLRP3 (AdipoGen; cryo-2), mouse-ASC (AdipoGen; AL177), pro and mature mouse-IL-1β (R&D Systems; AF-401-NA), pro and cleaved mouse caspase-1 (Santa Cruz; sc- 514) (Adipogen; AG-20B-0042-C100), and pro and cleaved mouse-GSDMD (Abcam; ab209845). Relevant horseradish peroxidase-conjugated secondary antibodies were applied for at least 1 h. Membranes were washed four times in TBS containing 0.1% (v/v) Tween 20 (TBST) between antibody incubations. Antibodies were diluted in TBST containing 5% skim milk. Immunoblots were developed using an ECL kit (Bio-Rad) in an ODYSSEY Fc (Li-COR) equipped with Image Lab software. All images were cropped for presentation; Full-size images will be presented upon request.

### Statistics

Data are presented as the mean ± standard deviation (SD). Comparisons were performed using RM one-way ANOVA, followed by Sidak's multiple comparison test, or RM two-way ANOVA, followed by Tukey's multiple comparison test. For each test, *P* values <0.05 were considered statistically significant.

## Results

### Accelerated VPRH-induced cell death in primary macrophages

**Figure 1.**
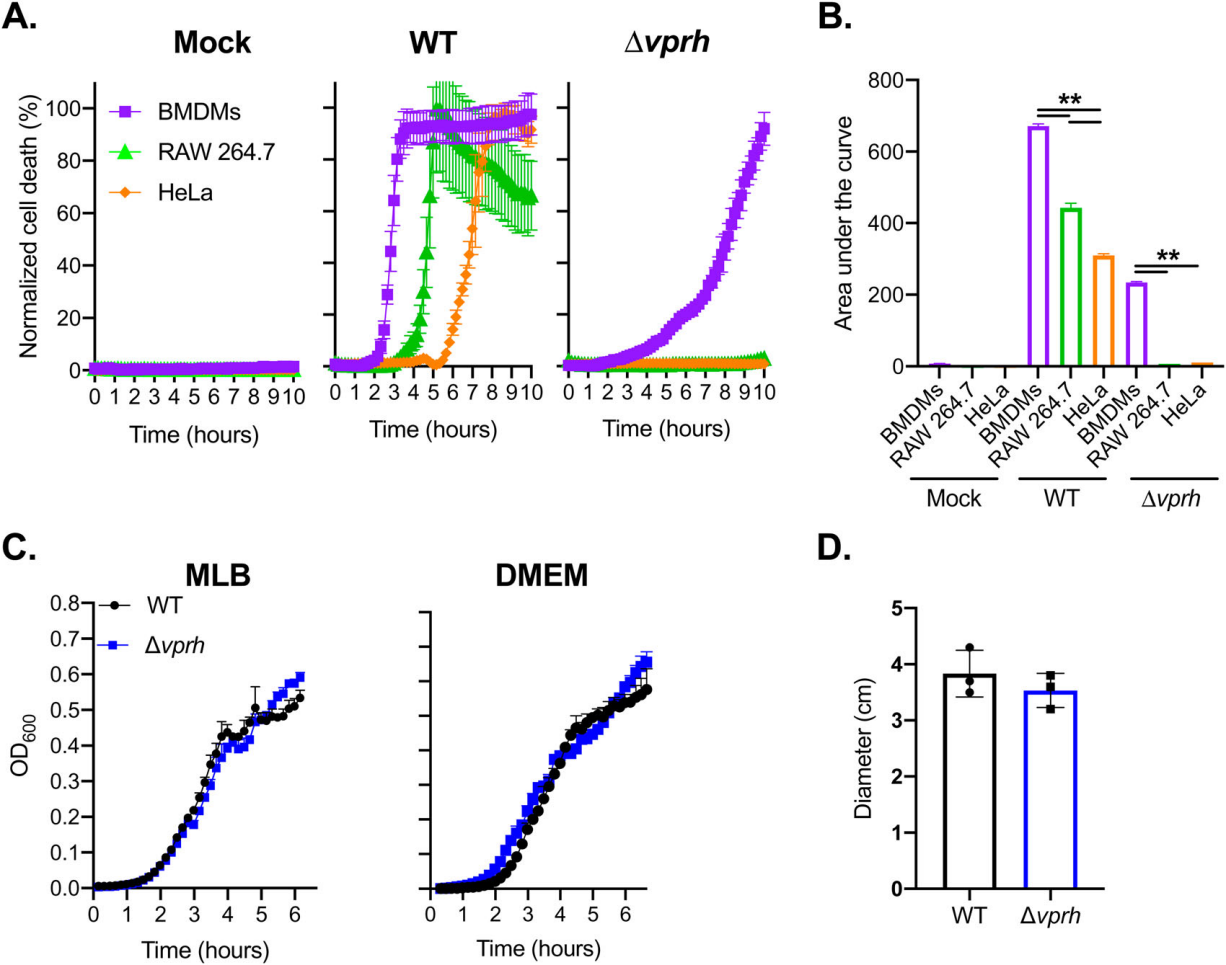
VPRH induces rapid cell death in primary macrophages. **(A)** Assessment of cell death upon infection of mammalian cells with *V. proteolyticus* strains. Approximately 3.5*10^4^ HeLa, RAW 264.7, or BMDM cells were seeded into 96-well plates in triplicate, and were infected with wild-type (WT) *V. proteolyticus* or a mutant in which we deleted *vprh* (Δ*vprh*), at the multiplicity of infection (MOI) 20. Propidium iodide (PI) was added to the medium prior to infection, and its uptake was assessed using real-time microscopy (IncucyteZOOM) and then graphed as the percentage of PI-positive cells normalized to the number of cells in the wells. **(B)** Summary of area under the curve (AUC) for results shown in panel A. Statistical comparisons between the different bacterial mutants and cell lines were carried using RM two-way ANOVA, followed by Tukey's multiple comparison test; the results are presented as the mean (bars) ± SD; *n* = 3; significant differences are denoted only for the comparison between cell lines in each infected strain; ** *P* *<* 0.01. **(C)** Growth of *V. proteolyticus* strains. Growth of *V. proteolyticus* strains, used in A, in MLB or DMEM media at 30°C, measured as absorbance at 600 nm (OD_600_). **(D)** Motility of *V. proteolyticus* strains. Swimming motility of *V. proteolyticus* strains, used in A, measured as migration on a soft-agar plate after overnight incubation at 30°C. The data in A, B, C and D are presented as the mean ± SD; *n* = 3. Results shown are representative of 3 independent experiments.

We previously reported that the pore-forming hemolysin, VPRH, induced actin cytoskeleton rearrangement and lysis in HeLa and RAW 264.7 cells upon infection with *V. proteolyticus* [11]. In this work, we sought to determine the effect of VPRH on primary immune cells. To this end, we repeated our original experiments, but with the addition of BMDMs, a known model for immune response cells, and a first-line defence against foreign invaders [22]. Importantly, BMDMs are also known to contain a full set of cell death mechanisms, including pyroptosis and necroptosis, as opposed to HeLa and RAW 264.7 cells [8,22]. As shown in Figure 1A, infection of HeLa and RAW 264.7 cells, as well as BMDMs resulted in VPRH-mediated induction of cell death, as evident by detection of propidium iodide (PI) uptake by cells (PI enters the cells and binds nucleic acids only upon the loss of membrane integrity). Interestingly, the VPRH-mediated cell death was more rapid in BMDMs (as can be seen by comparison of the area under the curve, AUC), suggesting that they are more sensitive than the previously tested HeLa and RAW 264.7 cells (Figure 1B). This result implies that cell death mechanisms that are missing in both the HeLa and RAW 264.7 cell lines may play a role in the rapid VPRH-induced cell death observed upon BMDM infection. Notably, a slower, VPRH-independent cell death was observed only in BMDMs upon infection with the *V. proteolyticus* Δ*vprh* mutant (Figure 1A,B).

The apparent cell death phenotypes in BMDMs were not a result of a fitness difference between the wild-type (WT) *V. proteolyticus* and the Δ*vprh* mutant strain, since we did not detect any difference in growth (either in MLB, the bacterial growth medium, or in DMEM, the medium in which infections were performed) (Figure 1C). Moreover, we tested bacterial swimming motility as an indication of fitness, since changes in motility may affect infection efficiency. As can be seen in Figure 1D, no significant difference in bacterial motility was observed, as determined by the diameter of bacterial migration in a swimming assay. Taken together, our results indicate that *V. proteolyticus* induces rapid cell death in BMDMs; this cell death is dependent on the pore-forming toxin, VPRH.

### VPRH-induced cell death in BMDMs is contact-independent

**Figure 2.**
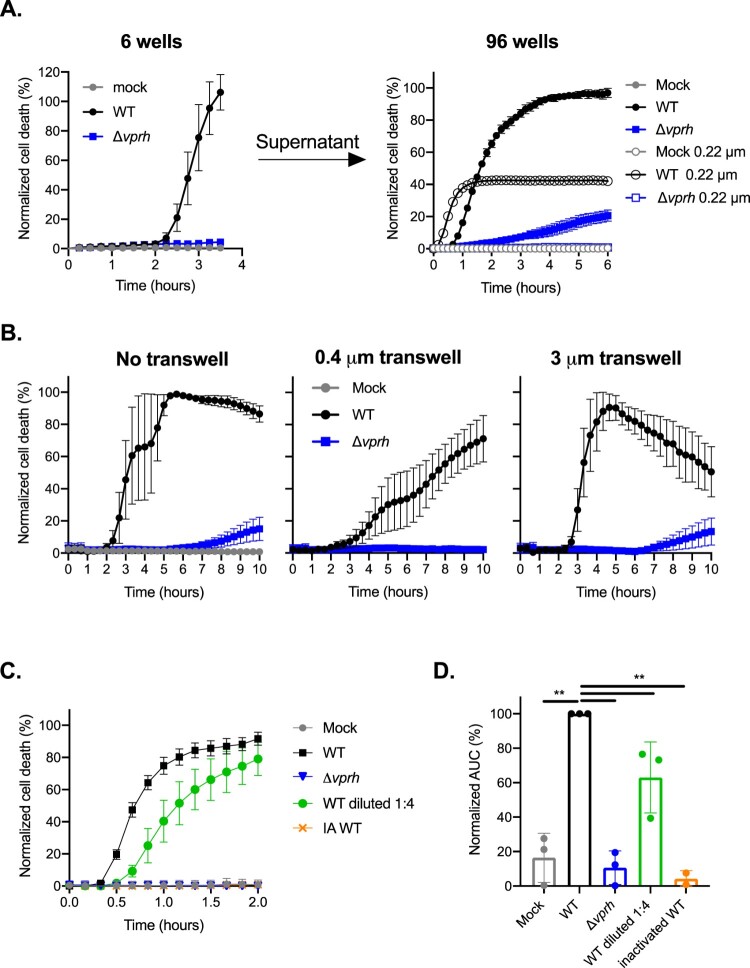
VPRH-mediated cell death is contact independent. **(A)** Approximately 2*10^6^ wild-type BMDMs were seeded into 6-well plates, and were infected with wild-type (WT) *V. proteolyticus* or a Δ*vprh* mutant at MOI 20. Supernatants collected 3.5 h post-infection were either filtered (0.22 μm filter) or not. Unfiltered or filtered supernatants were added in triplicate to BMDMs in a 96-well plate (3.5*10^4^ BMDMs per well). **(B)** *V. proteolyticus*, as in A, were added to cultures of 10^5^ BMDMs on top of 0.4 μm or 3 μm transwell filters, or without a transwell, in a 24-well plate. **(C)** Filtered supernatants from WT or Δ*vprh V. proteolyticus* cultures grown overnight in DMEM were added in triplicate to WT BMDMs in a 96-well plate (3.5*10^4^ BMDMs per well). In A, B and C, cell death was determined by monitoring propidium iodide (PI) uptake using real-time microscopy (IncucyteZOOM) and then graphed as the percentage of PI-positive cells normalized to the number of cells in wells. IA, inactivated; The data are presented as the mean ± SD; *n* = 3. Results shown are representative of 3 independent experiments. **(D)** Summary of normalized AUC for three independent biological experiments shown in panel C. Statistical comparisons between the different bacterial mutants were carried using RM one-way ANOVA, followed by Sidak's multiple comparison test; the results are presented as the mean (bars) ± SD of 3 independent experiments; significant differences are denoted only for the comparison between WT supernatant-treated BMDMs and other treatments; ** *P* < 0.01.

Bacterial-induced cell death may be either dependent or independent of contact with host cells. To test the hypothesis that VPRH-induced cell death in BMDMs is contact-independent, as we have shown previously in RAW 264.7 and HeLa cells [11], we eliminated any direct contact between the bacteria and BMDMs using two approaches. In the first approach, we infected BMDMs with *V. proteolyticus* strains for 3.5 h in 6-well culture plates while monitoring PI uptake (to measure cell death). Supernatants were collected from these wells, filtered (0.22 μm filter) to eliminate live bacteria, and then added to untreated BMDMs seeded in 96-well plates. Unfiltered supernatants from the same wells (containing live bacteria) were used as controls. As shown in Figure 2A, the supernatant of the WT-infected wells was still able to induce BMDM cell death, even after filtration (the black open circles), indicating that death was induced by a secreted protein present in the media. In the second approach, we physically separated the bacteria from the BMDMs using 0.4 μm transwells, and monitored cell death using PI uptake measurements (Figure 2B). Even when physically separated, the bacteria induced VPRH-dependent cell death in BMDMs, as they did when no transwells were used, or when 3 μm transwells (permeable to *V. proteolyticus* bacteria) were used. Taken together, these results suggest that VPRH-mediated cell death is caused by a secreted toxin in a contact-independent manner.

Another explanation for these results may be that VPRH induces the release of DAMPs, which then act in an autocrine manner to kill host cells. To eliminate any possibility of DAMPs release from dying cells, we collected the supernatants from bacteria that were grown overnight in DMEM, and added it directly to BMDMs. As can be seen by the kinetics of cell death and by the accompanying AUC analysis, only supernatants from WT *V. proteolyticus,* but not Δ*vprh* mutant strain, killed BMDMs (Figure 2C,D). This VPRH-dependent cell death was dose–dependent, as dilution of the WT supernatant slowed the killing kinetics down. Finally, we confirmed that this killing is dependent on protein in the supernatant, since denaturation of WT supernatant (95°C for 10 min) resulted in loss of killing activity (Figure 2C,D). Collectively, these findings demonstrate that the rapid cell death apparent in BMDMs is not due to DAMPs release from dying cells, but rather is directly mediated by VPRH in a contact-independent manner.

### VPRH induces a rapid, inflammasome-dependent pyroptotic cell death in BMDMs

**Figure 3.**
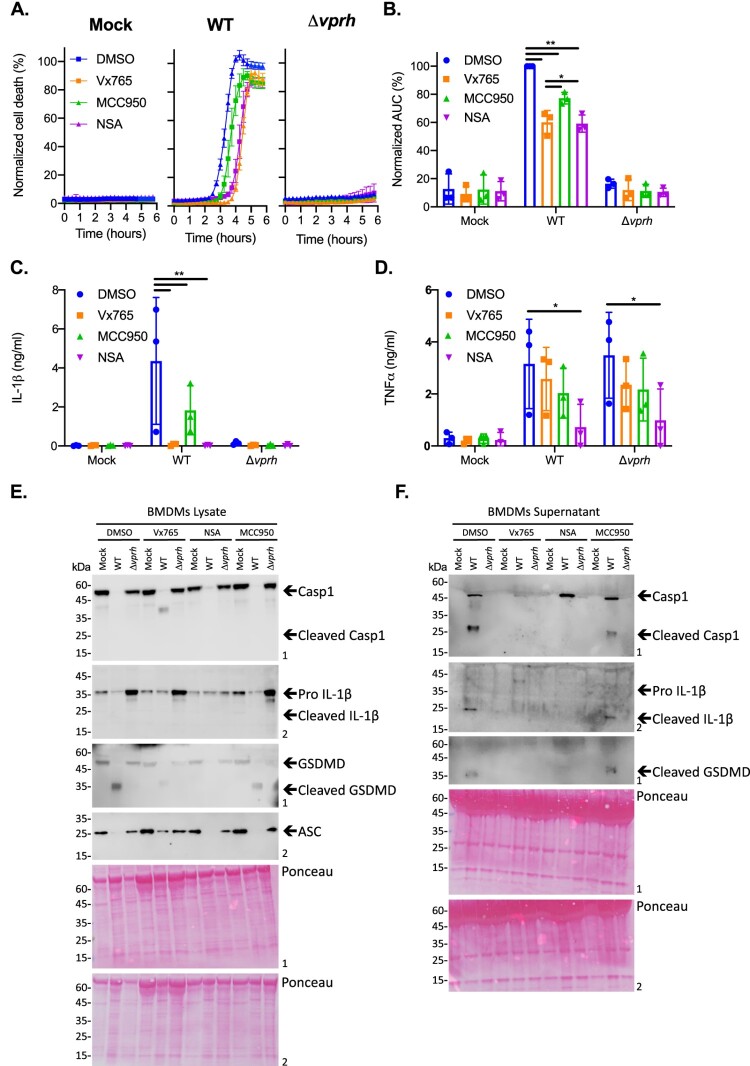
Inflammasome inhibitors delay VPRH-mediated cell death. Approximately 3.5*10^4^ wild-type BMDMs were seeded into 96-well plates in triplicate, and were primed using LPS (100 ng/ml) for 3 h prior to infection with *V. proteolyticus* mutants at MOI 50. Where indicated, inflammasome inhibitors – Vx765 (25 μM), MCC950 (20 μM), or NSA (20 μM), with the addition of propidium iodide (PI) (1 μg/ml), were added to the cells 30 min prior to bacterial infection. DMSO was added as the solvent control. **(A)** PI uptake was assessed using real-time microscopy (IncucyteZOOM) and then graphed as the percentage of PI-positive cells normalized to the number of cells in wells. Data are presented as the mean ± SD; *n* = 3. **(B)** Summary of normalized AUC for three independent biological experiments shown in panel A. **(C-E)** Cell lysates and supernatants from experiments described in A were collected 6 h post-infection. **(C, D)** IL-1β (C) and TNFα (D) secretion were measured using commercial ELISA kits. **(E, F)** Inflammasome components, caspase-1 (Casp1), GSDMD, and IL-1β cleavage were detected in BMDM lysates (E) and supernatants (F) using immunoblots (the numbers on the right of each blot indicate the blot number). The data in A, E, and F are representative of 3 independent experiments. Statistical comparisons in B, C and D between the different bacterial mutants and inflammasome inhibitors were carried using RM two-way ANOVA, followed by Tukey's multiple comparison test; the results are presented as the mean (bars) ± SD of 3 independent experiments; significant differences are denoted only for comparisons between inhibitors in each infected strain; * *P* *<* 0.05, ** *P* *< *0.01.

Since hemolysins were previously shown to induce inflammasome-mediated cell death [23], we hypothesized that the rapid VPRH-mediated cell death in BMDMs was also dependent on inflammasome activation. To test this, we added specific inflammasome inhibitors to BMDM cultures 30 min prior to infection with *V. proteolyticus* and monitored their effect on VPRH-induced cell death. The inhibitors used were as follows: (i) MCC950, which blocks ASC oligomerization by inhibiting the canonical and non-canonical NLRP3 inflammasome [24]; (ii) Vx765, a potent and selective competitive inhibitor of caspase-1 and -4 [25]; and (iii) Necrosulfonamide (NSA), a human specific mixed lineage kinase domain-like (MLKL) inhibitor that does not bind to the murine version of MLKL. When applied to murine cells, NSA blocks inflammasome priming, caspase-1 activation, and gasdermin D (GSDMD) pore formation [26]. DMSO was used as the solvent control. As shown in Figure 3A,B, the addition of NSA or Vx765, both inhibitors of caspase-1 activity, resulted in almost a two-hour delay of the VPRH-mediated cell death, and ∼40% reduction in the calculated AUC; the addition of MCC950, which specifically inhibits the NLRP3 inflammasome, resulted in a shorter, one-hour delay of cell death, and ∼25% reduction in the calculated AUC. Taking the protective effect of the three independent inflammasome pathway inhibitors against infection with WT *V. proteolyticus*, we hypothesized that VPRH induces a rapid cell death in BMDMs via the inflammasome-dependent pyroptotic pathway. Since a delayed cell death was still evident, even in the presence of the inflammasome inhibitors, we also hypothesized that a second step, which is inflammasome-independent, plays a role in the VPRH-induced cell death in BMDMs.

To further support our hypothesis that VPRH induces a rapid, inflammasome-mediated cell death in BMDMs, we sought to determine whether the inflammasome-dependent cytokine, IL-1β, was secreted upon infection with *V. proteolyticus*. To this end, we determined the amount of the cytokines IL-1β and TNFα (an NF-κB-dependent, inflammasome-independent cytokine that was used as a control) in the supernatants of the infected cultures described above (Figure 3A). In agreement with the above results, the addition of inflammasome inhibitors eliminated IL-1β secretion (Figure 3C), whereas Vx765 and MCC950 had no effect on TNFα secretion (Figure 3D). These results confirm that VPRH induces an inflammasome-dependent cell death in BMDMs.

We also determined the effect that inflammasome inhibitors had on VPRH-mediated, inflammasome-dependent cell death by monitoring the cleavage and release of caspase-1, IL-1β, and GSDMD in BMDMs infected with WT *V. proteolyticus*. As shown in Figure 3E,F, Vx765 and NSA, which resulted in a two-hour delay in cell death, also inhibited the various inflammasome-dependent phenotypes that were tested: (i) caspase-1 processing and release to the supernatant, (ii) IL-1β maturation and secretion, and (iii) GSDMD processing. Notably, MCC950, which had a milder effect on cell death, was also less effective in inhibiting these inflammasome-dependent phenotypes. Collectively, these findings suggest that the rapid cell death apparent in BMDMs is mediated by VPRH-induced activation of pyroptosis, possibly via the NLRP3 inflammasome.

### VPRH activates the NLRP3 inflammasome in BMDMs

Inflammasome activation may be induced by several NLR family members, including NLRP1 and NLRP3 [27]. The most studied inflammasome is NLRP3, which was shown to be activated by many DAMPs and PAMPs. Furthermore, NLRP3 was also shown to be activated by the necroptotic cell death pathway, which is induced by pseudo-kinase MLKL [28,29]. Thus, to identify the specific inflammasome pathway that was activated by VPRH in BMDMs, we cultured BMDMs from knockout (KO) mice in which different inflammasome activation pathways were severed (i.e. *Mlkl^-/-^*, *Nlrp1^-/-^*, and *Nlrp3^-/-^*). We used real-time microscopy to compare their cell death kinetics to those of BMDMs from WT mice during *V. proteolyticus* infection. We also monitored IL-1β secretion using ELISA, as well as by processing various inflammasome components to determine inflammasome activation in BMDMs from the KO mice.

**Figure 4.**
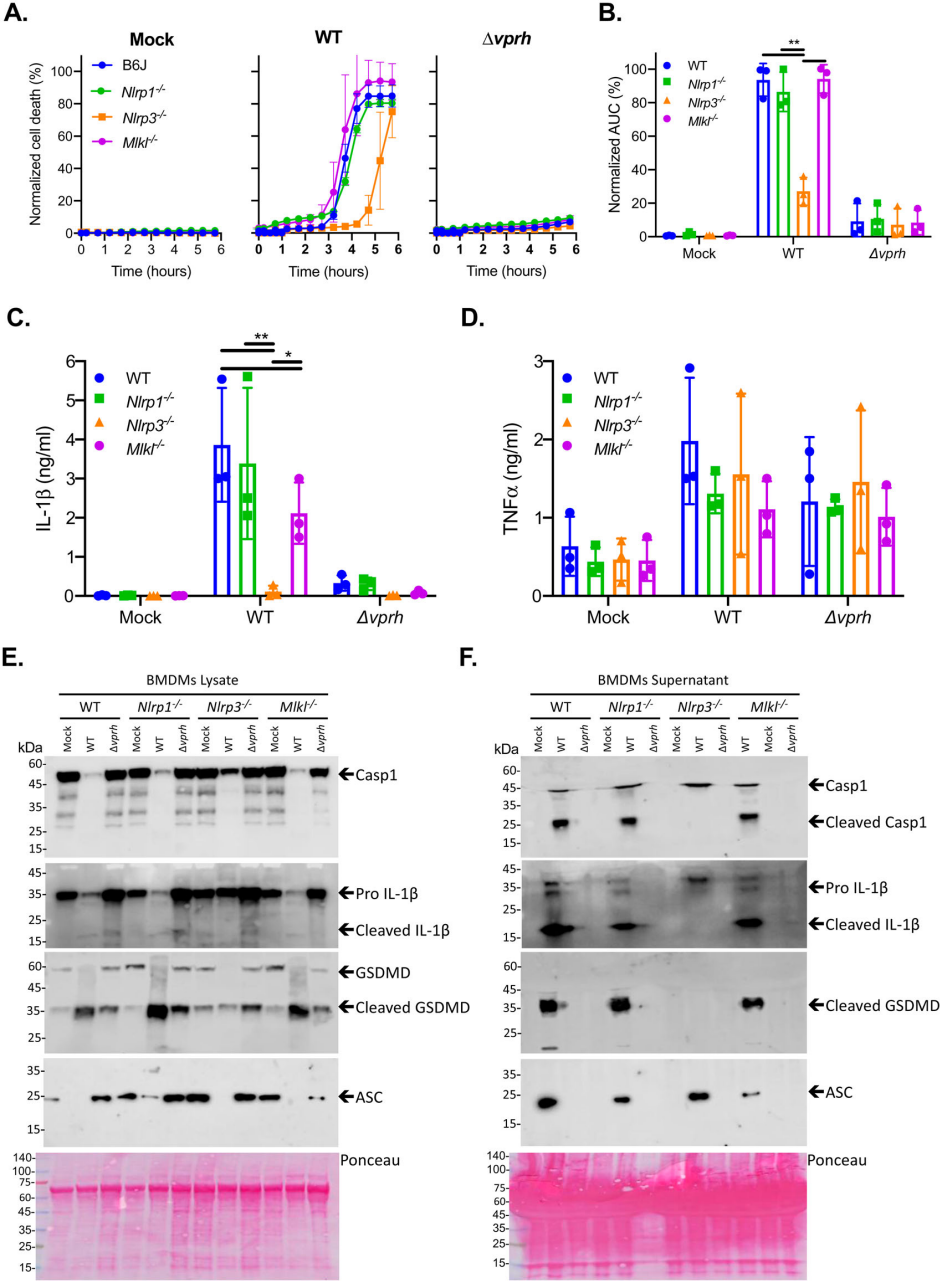
VPRH induces cell death via the NLRP3 inflammasome. Approximately 3.5*10^4^ wild-type (B6J), *Nlrp1^-/-^*, *Nlrp3^-/-^*, and *Mlkl^-/-^* BMDMs were seeded into 96-well plates in triplicate, and were primed using LPS (100 ng/ml) for 3 h prior to infection with *V. proteolyticus* mutants at MOI 20. **(A)** PI uptake was assessed using real-time microscopy (IncucyteZOOM) and then graphed as the percentage of PI-positive cells normalized to the number of cells in wells. Data are presented as the mean ± SD; *n* = 3. **(B)** Summary of normalized AUC for three independent biological experiments shown in panel A. **(C-F)** Cell lysates and supernatants from experiments described in A were collected 6 h post-infection. **(C, D)** IL-1β (C) and TNFα (D) secretion were measured using commercial ELISA kits. **(E, F)** Inflammasome components and caspase-1 (Casp1), GSDMD, and IL-1β cleavage were detected in BMDM lysates (E) and supernatants (F) using immunoblots (the numbers on the right of each blot indicate the blot number). The data in A, E, and F are representative of 3 independent experiments. Statistical comparisons in B, C and D between the different bacterial mutants and mouse strains were carried using RM two-way ANOVA, followed by Tukey's multiple comparison test; the results are presented as the mean (bars) ± SD of 3 independent experiments; significant differences are denoted only for comparisons between mouse strains in each infected strain; *P < 0.05, **P < 0.01.

As shown in Figure 4A, infection of BMDMs from *Nlrp1^-/-^* and *Mlkl^-/-^* mice resulted in rapid cell death, with kinetics comparable to those observed in BMDMs from WT (B6J) mice. Remarkably, infection of *Nlrp3^-/-^* BMDMs resulted in a delayed cell death phenotype (Figure 4A), similar to the phenotype observed when inflammasome inhibitors were added to WT BMDMs (Figure 3A). Statistical analysis of cell death kinetics using calculation of AUC confirmed that NLRP3 was responsible for the rapid cell death (Figure 4B). These results indicate that NLRP3, but not NLRP1 or MLKL, is required for the rapid cell death induced by VPRH in BMDMs. In agreement with these results, IL-1β secretion was abrogated specifically in *Nlrp3^-/-^* BMDMs (Figure 4C). In contrast, TNFα secretion was not affected in either of the KO mouse BMDMs (Figure 4D), thus confirming that priming (i.e. the NF-κB-dependent pathway) remained unaffected in the *Nlrp3^-/-^* BMDMs. In further support of the above results and conclusions, cleaved caspase-1, mature IL-1β, and cleaved GSDMD were absent in *Nlrp3^-/-^* BMDM culture lysates and supernatants (Figure 4E,F).

### Exogenous complementation of VPRH restores NLRP3-dependent cell death in BMDMs

**Figure 5.**
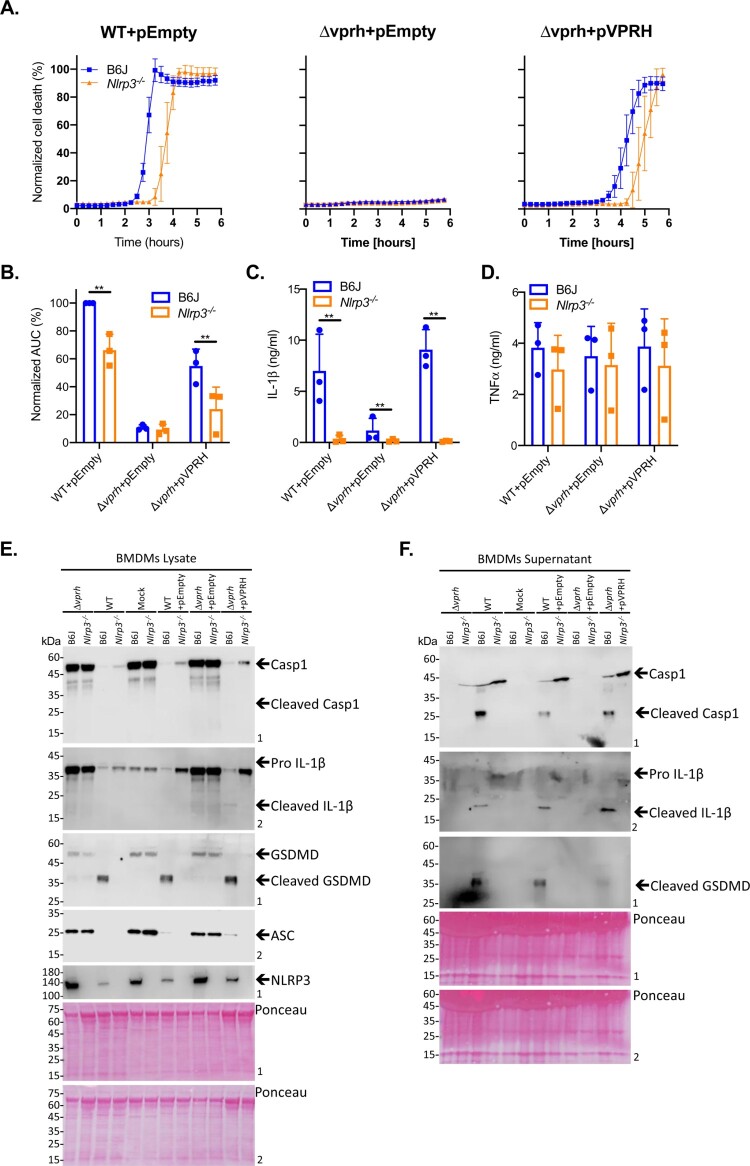
Exogenous complementation of VPRH restores the NLRP3 inflammasome activation in BMDMs. Approximately 3.5*10^4^ wild-type (B6J) and *Nlrp3^-/^*^-^ BMDMs were seeded into 96-well plates and then primed using LPS (100 ng/ml) for 3 h prior to infection with *V. proteolyticus* mutants at MOI 50. **(A)** PI uptake was assessed using real-time microscopy (IncucyteZOOM) and graphed as the percentage of PI-positive cells normalized to the number of cells in the wells. Data are presented as the mean ± SD; *n* = 3. **(B)** Summary of normalized AUC for three independent biological experiments as shown in panel A. **(C-F)** Cell lysates and supernatants from experiments described in A were collected 6 h post-infection. **(C, D)** IL-1β (C) and TNFα (D) secretion were measured using commercial ELISA kits. **(E, F)** Inflammasome components, caspase-1 (Casp1), GSDMD, and IL-1β cleavage were detected in BMDM lysates (E) and supernatants (F) using immunoblots (the numbers on the right of each blot indicate the blot number). The data in A, E, and F are representative of 3 independent experiments. Statistical comparisons in B, C and D between the different bacterial mutants and mouse strains were carried using RM two-way ANOVA, followed by Tukey's multiple comparison test; the results are presented as the mean (bars) ± SD of 3 independent experiments; significant differences are denoted only for comparisons between mouse strains in each infected strain; **P < 0.01.

To further confirm our hypothesis that VPRH induces NLRP3 inflammasome-dependent cell death in BMDMs, we introduced *vprh* on an arabinose-inducible expression plasmid (pVPRH) back into the Δ*vprh* strain. As shown in Figure 5, complementation of VPRH restored all of the inflammasome-mediated phenotypes upon BMDM infection, including rapid cell death (Figure 5A,B), IL-1β secretion (Figure 5C), as well as cleavage of caspase-1, maturation of IL-1β, and cleavage of GSDMD (Figure 5E,F). Notably, exogenous expression of VPRH had no effect on TNFα secretion (Figure 5D). Importantly, the phenotypes that were observed with the VPRH-complemented strain were NLRP3-dependent, as evident by the fact that none of them were restored when *Nlrp3^-/-^* BMDMs were infected (Figure 5).

### VPRH activates the NLRP3 inflammasome in human PBMCs

**Figure 6.**
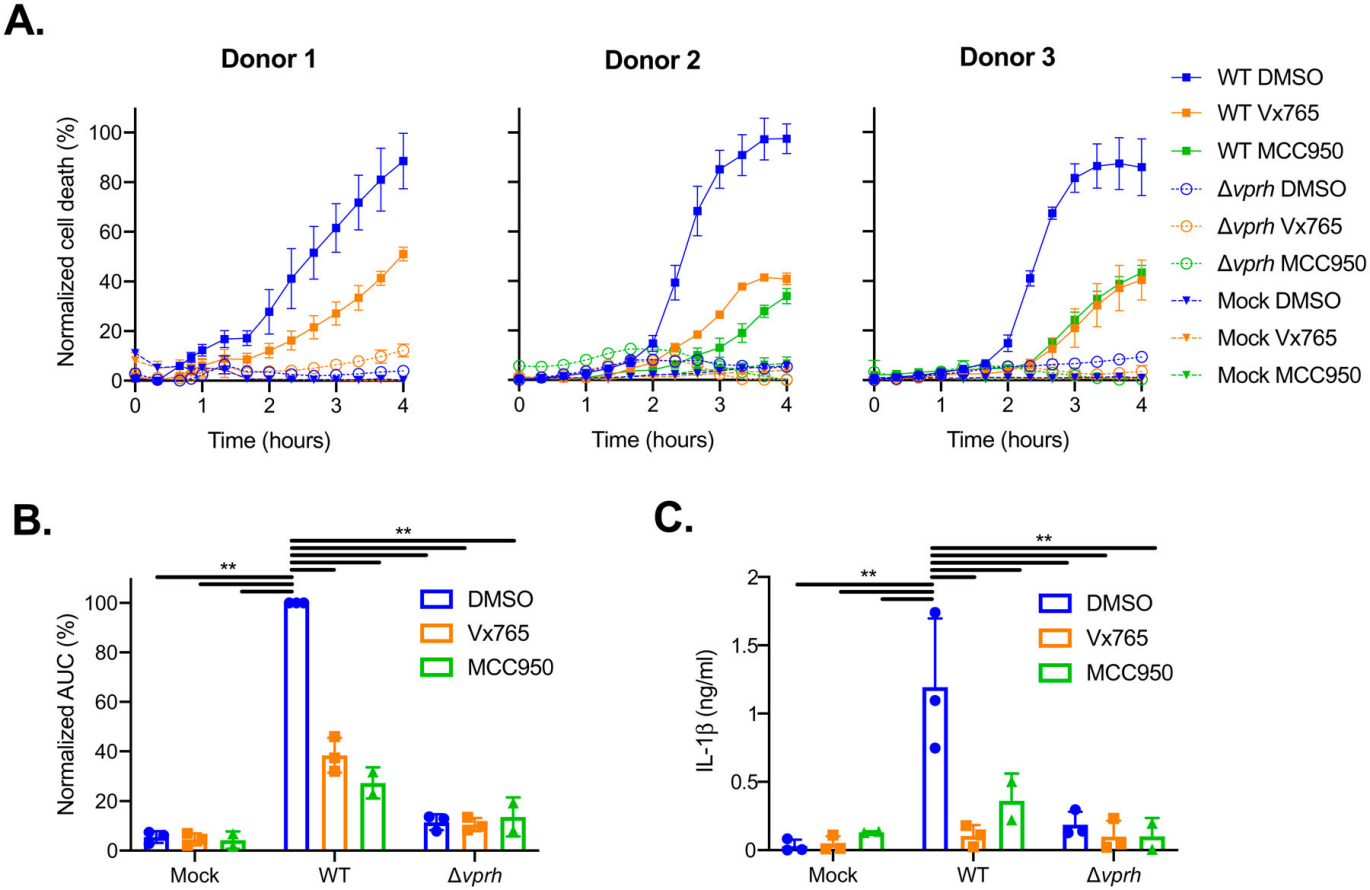
Inflammasome inhibitors delay VPRH-mediated cell death in human PBMCs. Approximately 2*10^4^ healthy donor PBMCs were seeded into 96-well plates in triplicate for 18 h prior to infection with *V. proteolyticus* mutants at MOI 50. Where indicated, inflammasome inhibitors – Vx765 (25 μM) or MCC950 (20 μM), with the addition of propidium iodide (PI) (1 μg/ml), were added to the cells 30 min prior to bacterial infection. DMSO was added as the solvent control. In donor 1, only the inhibitor VX765 was applied. **(A)** PI uptake was assessed using real-time microscopy (IncucyteZOOM) and then graphed as the percentage of PI-positive cells normalized to the number of cells in wells. Data are presented as the mean ± SD; *n* = 3. **(B)** Summary of normalized AUC for the three donors shown in panel A. **(C)** Supernatants from experiments described in A were collected 4 h post-infection, and IL-1β secretion was measured using commercial ELISA kit. Statistical comparisons in B and C between the different bacterial mutants and inflammasome inhibitors were carried using RM two-way ANOVA, followed by Tukey's multiple comparison test; the results are presented as the mean (bars) ± SD of 3 independent experiments; significant differences are denoted only for the comparisons between PBMCs infected with WT *V. proteolyticus* and the other condition; ** *P* < 0.01.

Next, we set out to determine whether *V. proteolyticus* may be an emergent concern for human health. To this end, we tested the effect of VPRH on human peripheral blood mononuclear cells (PBMCs) from healthy donors, since it is well known that human and mouse may differ in their sensitivity to inflammasome activation [10,30–32]. As shown in Figure 6A,B, infection of PBMCs from healthy human donors resulted in rapid cell death, with kinetics comparable to those observed in BMDMs. Remarkably, treatment of PBMCs with either caspase-1 or NLRP3 inhibitors resulted in a delayed cell death phenotype (Figure 6A,B), similar to the phenotype observed when inflammasome inhibitors were added to WT BMDMs (Figure 3A). These results indicate that NLRP3 inflammasome is required for the rapid cell death induced by VPRH in human PBMCs. In agreement with these results, IL-1β secretion was abrogated specifically when inflammasome inhibitors were used (Figure 6C). These results indicate that *V. proteolyticus* possess deleterious abilities towards human macrophages.

## Discussion

We previously showed that the *V. proteolyticus* VPRH, a leukocidin domain-containing hemolysin, induces cell death upon infection of HeLa and RAW 264.7 cells [11]. Here, we found that this cell death phenotype is accelerated when BMDMs are infected. Since BMDMs, unlike HeLa and RAW 264.7 cells, possess functional inflammasomes whose activation can lead to rapid cell death, we hypothesized that VPRH activates the inflammasome in BMDMs. Our results clearly indicate that in BMDMs, VPRH induces a two-step cell death. Although the mechanism leading to the second, slower step remains to be elucidated, we showed that the first, rapid step is mediated by VPRH-dependent activation of the inflammasome. It is plausible that the second, slower step of cell death observed in BMDMs is mediated by the same mechanism that induced VPRH-dependent cell death in HeLa and RAW 264.7 cells, which lack a functional inflammasome. Using a combination of genetic and chemical approaches, we identified the NLRP3 inflammasome as the specific pathway responsible for the VPRH-induced rapid cell death in BMDMs. Finally, using freshly isolated human PBMCs, we showed that this virulence mechanism is highly relevant to human pathology, and thus may be an emergent concern for human health. Taken together, our results shed new light on the virulence potential of VPRH, and possibly that of other members of this leukocidin class of pore-forming toxins. Although VPRH induces cell death in non-immune cells, such as HeLa cells, it can also specifically activate innate immune response mechanisms in primary macrophages by activating the NLRP3-inflammasome pathway. It remains to be investigated whether activation of NLRP3 inflammasome-mediated cell death benefits the pathogen or the host during infection.
